# Functional outcome of traumatic spinopelvic instabilities treated with lumbopelvic fixation

**DOI:** 10.1038/s41598-020-71498-6

**Published:** 2020-09-10

**Authors:** Emre Yilmaz, Martin F. Hoffmann, Alexander von Glinski, Christiane Kruppa, Uwe Hamsen, Cameron K. Schmidt, Ahmet Oernek, Matthias Koenigshausen, Marcel Dudda, Thomas A. Schildhauer

**Affiliations:** 1 Department of General and Trauma Surgery, BG University Hospital Bergmannsheil, Buerkle-de-la-Camp Platz 1, Bochum, NRW Germany; 2 Swedish Neuroscience Institute, Swedish Medical Center, Seattle, Washington USA; 3 Institute of Diagnostic and Interventional Radiology and Nuclear Medicine, BG-University-Hospital Bergmannsheil, Bochum, Germany; 4 Department of Trauma Surgery, University Hospital Essen, Essen, Germany; 5 Ruhr University Bochum, Bochum, Germany

**Keywords:** Orthopaedics, Outcomes research

## Abstract

The aim of this study was to assess the functional outcome after lumbopelvic fixation (LPF) using the SMFA (short musculoskeletal functional assessment) score and discuss the results in the context of the existing literature. The last consecutive 50 patients who underwent a LPF from January 1st 2011 to December 31st 2014 were identified and administered the SMFA-questionnaire. Inclusion criteria were: (1) patient underwent LPF at our institution, (2) complete medical records, (3) minimum follow-up of 12 months. Out of the 50 recipients, 22 questionnaires were returned. Five questionnaires were incomplete and therefore seventeen were included for analysis. The mean age was 60.3 years (32–86 years; 9m/8f) and the follow-up averaged 26.9 months (14–48 months). Six patients (35.3%) suffered from a low-energy trauma and 11 patients (64.7%) suffered a high-energy trauma. Patients in the low-energy group were significantly older compared to patients in the high-energy group (72.2 vs. 53.8 years; *p* = 0.030). Five patients (29.4%) suffered from multiple injuries. Compared to patients with low-energy trauma, patients suffering from high-energy trauma showed significantly lower scores in “daily activities” (89.6 vs. 57.1; *p* = 0.031), “mobility” (84.7 vs. 45.5; *p* = 0.015) and “function” (74.9 vs. 43.4; *p* = 0.020). Our results suggest that patients with older age and those with concomitant injuries show a greater impairment according to the SMFA score. Even though mostly favorable functional outcomes were reported throughout the literature, patients still show some level of impairment and do not reach normative data at final follow-up.

## Introduction

The surgical treatment of lumbosacral instabilities remains a challenge to this date. Several surgical options, including the S2 alar iliac (S2AI) screw, iliosacral screws, sacral bars and sacral plates have been developed since the Galveston technique was first described by Allen and Ferguson^1,2^. In 1994 the lumbopelvic fixation (LPF) was introduced by Kaech and Tranz as a combination of horizontal and vertical osteosynthesis and was later modified by Schildhauer et al.^3,4^. Since its first description, the lumbopelvic fixation technique has been used to treat instabilities of the lumbopelvic region and is widely established. The technique has been analyzed with regards to safety, reliability, intra- and postoperative complications, radiographical, and neurological outcome. Lumbopelvic fixation has been proven to provide high stability, allowing early weight-bearing and making this technique useful in high-grade instabilities, deformity fixations, sacral tumor resections, and displaced fracture fixation. On the other hand, the technique is criticized for its high rate of wound complications and infections^5–7^. However, the literature is lacking in studies reporting the functional outcome of patients who underwent lumbopelvic fixation. We therefore assessed the functional outcome after lumbopelvic fixation using the SMFA (short musculoskeletal functional assessment) score and performed a literature review.

## Patients and methods

**Figure 1:**
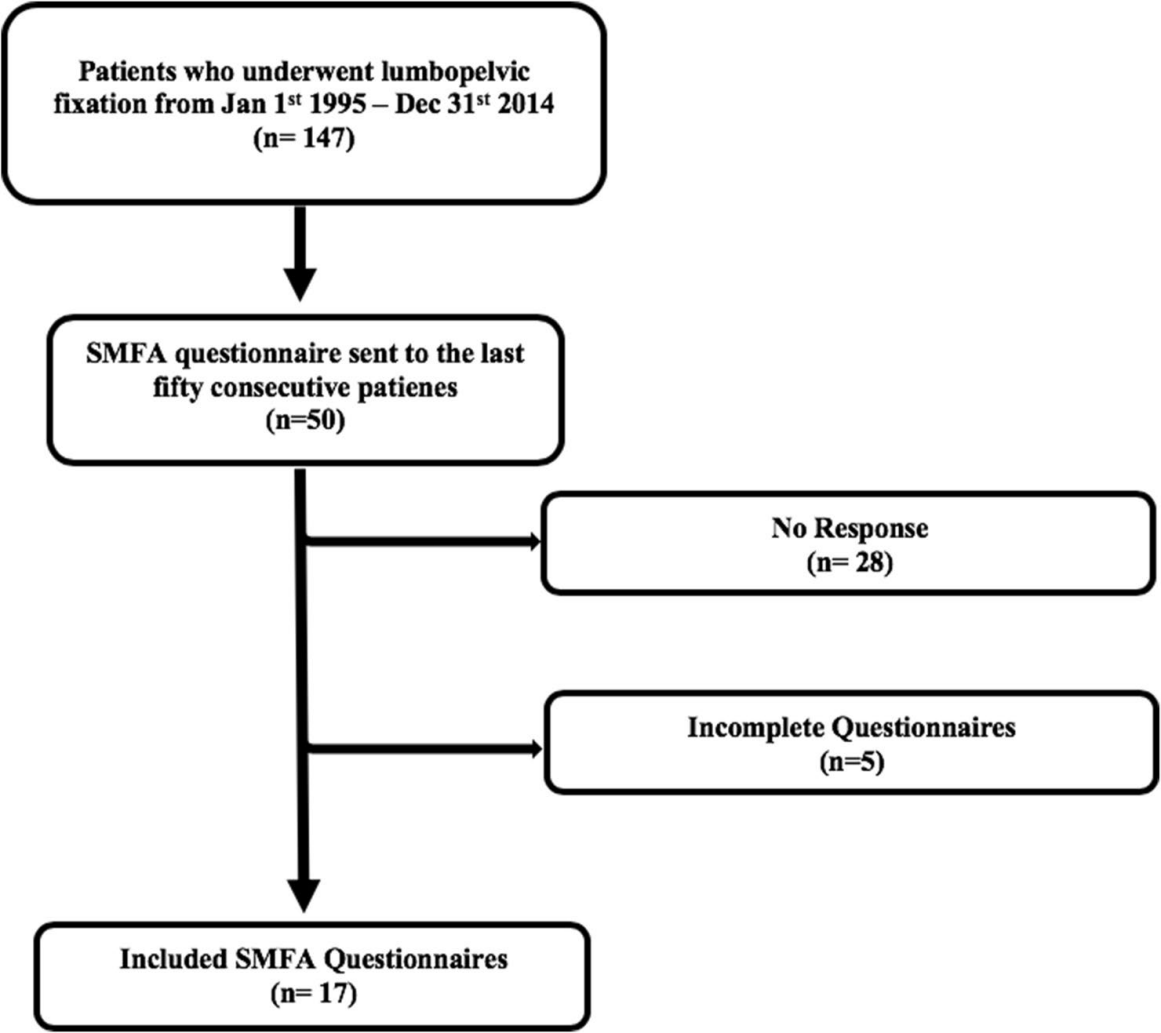
Flow diagram (SMFA questionnaires).

This study has been approved by the local ethics committee of the Ruhr-University Bochum (No. 16-5711-BR). All methods were performed in accordance with the relevant guidelines and regulations. Written informed consent was obtained from all patients. The last consecutive 50 patients who underwent a LPF from January 1st 2011 to December 31st 2014 were identified and administered the SMFA questionnaire. Inclusion criteria were: (1) patient underwent LPF at our institution, (2) complete medical records, (3) minimum follow-up of 12 months. Out of the 50 recipients, 22 questionnaires were returned. Five questionnaires were incomplete and therefore seventeen were included for analysis (Fig. 1). The following data were ascertained from the patient’s medical records: gender, age, etiology, associated injuries, American Society of Anaesthesiologists’ (ASA) classification, level of surgery, type of surgery, complications and trauma mechanism (low- vs. high-energy trauma). Low-energy trauma was defined as a result of falling from standing height or low height less than 1 m, while high-energy trauma was defined as any other type of trauma (e.g. motor vehicle accident or falling from heights).

### SMFA questionnaire

The SMFA questionnaire is a patient-based survey that has been demonstrated to be a valid and reliable tool in the assessment of functional impairments. The questionnaire consists of 46 items within two main groups (functional and bother index) that assess functional impairment over the last week. The functional index represents daily activities, emotional status, mobility and arm-hand function. Higher scores indicate a greater degree of dysfunction or bother^8,9^.

### Statistical analysis

Univariate analysis was performed to compare demographics, surgical characteristics, complications, and SMFA scores. To assess for statistical differences between groups an unpaired Students t-test was used when appropriate. Fisher’s exact test was performed to determine the significance of categorial data. For continuous outcomes, simple linear regression was used. Statistical significance was set at *p* < 0.05. Data were analyzed using SPSS version 22.0 (SSPS Inc, Chicago, IL).

### Ethical approval

The study has been approved by the local Ethical Committee.

## Results

In our patient population, the mean age was 60.3 years (32–86 years). Nine males (52.9%) and 8 females (47.1%) fulfilled the inclusion criteria. Follow-up averaged 26.9 months (14–48 months). Six patients (35.3%) suffered a low-energy trauma (ground-level fall or fall < 1 m) and 11 patients (64.7%) suffered a high-energy trauma (traffic accident or fall > 1 m). Patients in the low-energy group were significantly older compared to patients in the high-energy group (72.2 vs. 53.8 years; *p* = 0,030). Five patients (29.4%) suffered multiple injuries, fractures of the lower extremities in two cases, one fractured acetabulum, one traumatic brain injury, and one vertebral fracture. The vast majority of patients underwent a LPF from L5 to pelvis (82.4%). A bilateral LPF was performed in 12 patients (70.6%) (Table 1). Postoperative complications occurred in five cases including three wound healing complications.

**Table 1:** Overview SMFA score results.

		Daily activities	Emotional	Arm/hand	Mobility	Function	Bother
	n (%)	Mean (±SD)					
**Gender**							
Male	9 (52.9%)	56.4 (11.9)	46.8 (9.1)	31.6 (9.7)	47.5 (12.7)	46.2 (10.5)	53.0 (11.1)
Female	8 (47.1%)	82.2 (5.9) *p* = 0.82	54.0 (6.3) *p* = 0.536	40.6 (15.5) *p* = 0.588	72.6 (8.2) *p* = 0.127	63.9 (7.5) *p* = 0.199	64.8 (6.7) *p* = 0.389
**Trauma mechanism**							
Low energy	6 (35.3%)	**89.6 (5.9)**	63.7 (5.8)	55.2 (15.9)	**84.7 (5.5)**	**74.9 (7.0)**	72.2 (7.6)
High energy	11 (64.7%)	**57.1 (9.4) p = 0.031**	42.9 (7.2) *p* = 0.071	25.3 (7.5) *p* = 0.071	**45.5 (10.0)** ***p = 0.015***	**43.4 (8.0)** ***p = 0.02***	51.1 (8.8) *p* = 0.131
**Etiology**							
Fall	13 (76.5%)	71.5 (8.0)	51.6 (7.0)	35.1 (9.5)	63.0 (8.5)	56.3 (7.5)	59.6 (7.3)
Traffic accident	4 (23.5%)	60.0 (19.7) *p* = 0.541	45.5 (7.8) *p* = 0.656	38.3 (16.6) *p* = 0.872	47.2 (22.2) *p* = 0.426	48.5 (16.7) *p* = 0.639	55.2 (15.1) *p* = 0.788
**Ass. injury**							
None	12 (70.6%)	61.25 (9.5)	**41.1 (5.8)**	27.4 (9.6)	**50.7 (10.5)**	**46.1 (8.3)**	**50.0 (7.9)**
Any ass. injury	5 (29,4%)	86.00 (6.5) *p* = 0.48	**72.14 (5.5)** ***p* = 0.002**	56.3 (10.1) *p* = 0.63	**80.0 (3.9)** ***p* = 0.021**	**74.6 (4.4)** ***p* = 0.009**	**79.2 (3.1)** ***p* = 0.009**
Lower extremity	2 (11.8%)	75.0 (12.5)	80.4 (1.8)	35.9 (14.1)	75.0 (2.8)	66.9 (8.1)	86.5 (3.1)
Acetabular fx	1 (5.9%)	95.0	82.1	78.1	80.6	84.6	64.6
Traumatic brain injury	1 (5.9%)	85.0	57.1	75.0	75.0	74.3	66.7
Vertebral fracture	1 (5.9%)	100.0	60.7	56.3	94.4	80.2	91.7
**ASA**							
1	5 (29.4%)	41.0 (15.4)	39.3 (13.0)	23.1 (13.6)	36.7 (15.5)	35.1 (13.6)	41.3 (15.0)
2	6 (35.3%)	77.1 (10.6)	55.4 (9.1)	49.8 (18.3)	62.5 (15.5)	62.2 (12.7)	57.6 (7.8)
3	5 (29.4%)	79.5 (7.1)	52.9 (9.1)	28.1 (6.6)	71.7 (8.2)	59.4 (6.3)	70.4 (10.6)
4	1 (5.9%)	100.0	60.71	56.3	94.4	80.2	91.7
**Level of surgery**							
L4 to pelvis	3 (17.6%)	65.0 (17.7)	42.9 (14.4)	38.5 (31.1)	48.1 (24.7)	49.5 (22.0)	52.8 (14.6)
L5 to pelvis	14 (82.4%)	69.3 (8.5) *p* = 0.841	51.8 (6.2) *p* = 0.612	35.3 (7.9) *p* = 0.927	61.7 (8.7) *p* = 0.646	55.6 (7.2) *p* = 0.814	59.8 (7.6) *p* = 0.695
**Surgery**							
Bilateral LPF	5 (29.4%)	76.5 (19.3)	60.0 (14.7)	48.1 (16.9)	69.4 (17.8)	64.6 (16.2)	65.0 (17.0)
LPF bilateral + Lam	5 (29.4%)	70.5 (10.4)	40.7 (8.2)	26.9 (18.5)	56.1 (14.7)	49.8 (12.1)	50.0 (8.4)
Lpf bilateral + Spon	1 (5.9%)	62.5	78.6	21.9	72.2	58.8	83.3
Lpf + ISF bilateral	1 (5.9%)	85.0	57.1	75.0	75.0	74.3	66.7
Lpf + ISF unilateral	3 (17.6%)	69.2 (17.3)	48.8 (8.6)	35.4 (11.0)	53.7 (26.9)	52.9 (16.4)	69.4 (15.5)
Lpf unilteral	2 (11.8%)	37.5 (22.5)	33.9 (1.8)	15.6 (12.5)	36.1 (19.5)	31.3 (15.1)	31.3 (8.3)
**Complications**							
None	12 (70.6%)	62.9 (9.6)	46.1 (6.6)	34.6 (9.9)	54.2 (10.4)	50.3 (8.5)	55.6 (8.3)
Any complication	5 (24.9%)	82.0 (8.5) *p* = 0.161	60.0 (10.1) *p* = 0.285	38.8 (14.5) *p* = 0.823	71.6 (11.4) *p* = 0.281	64.5 (10.3) *p* = 0.311	65.8 (11.0) *p* = 0.475
Incomplete Cauda syndrome	1 (5.9%)	95.0	82.1	78.1	80.6	84.6	64.6
Perforated drain	1 (5.9%)	52.5	32.1	0	27.8	29.4	43.8
Wound healing complication	3 (17.6%)	87.5 (7.2)	61.9 (11.4)	38.5 (14.7)	83.3 (5.6)	69.6 (8.1)	73.6 (17.0)

p < 0.05 values are indicated in bold

*Ass.* associated, *Fx* fracture, *LPF* lumbopelvic fixation, *Lam*: laminectomy, *Spon* spondylodesis, *ISF* iliosacral screw fixation.

Analyzing the SMFA score, no significant gender-related differences were found in daily activities (56.4 vs. 82.2; *p* = 0.82), emotional score (46.8 vs. 54.0; *p* = 0.536), arm/hand score (31.6 vs. 40.6; *p* = 0.588), mobility (47.5 vs. 72.6; *p* = 0.127), function (46.2 vs. 63.9; *p* = 0.199) and bother score (53.9 vs. 64.8; *p* = 0.389).

Compared to patients with low-energy trauma, patients suffering from high-energy trauma showed significantly lower scores in “daily activities” (89.6 vs. 57.1; *p* = 0.031), “mobility” (84.7 vs. 45.5; *p* = 0.015) and “function” (74.9 vs. 43.4; *p* = 0.02). Patients with a concomitant injury had significantly higher scores in “emotional” (41.1 vs. 72.1; *p* = 0.002), “mobility” (50.7 vs. 80.0; *p* = 0.021), “function” (46.1 vs. 74.6; *p* = 0.009) and “bother” (50.0 vs. 79.2; *p* = 0.009). Patients with higher ASA score showed higher SMFA scores throughout all sub-scores. No significant differences in SMFA scores were found with respect to etiology (fall vs. motor vehicle accident), operative treatment, and complications (Table 1).

### Literature review

The search term “lumbopelvic fixation or triangular osteosynthesis or spinopelvic fixation” was used in the database PubMed (access date: 06/20/20). Studies reporting the following functional outcome scores were included: SMFA, SF-36, EuroQol-6D, EQ-5D, ODI, Majeed, Hannover Pelvis Outcome Score, VAS, and/or a description of the neurological outcome. Exclusion criteria were as follows: (1) not reporting LPF, (2) biomechanical, anatomical, technical report (3) not trauma-related, (4) reviews and case reports, (5) article not available or (6) no functional outcome was reported.

**Figure 2:**
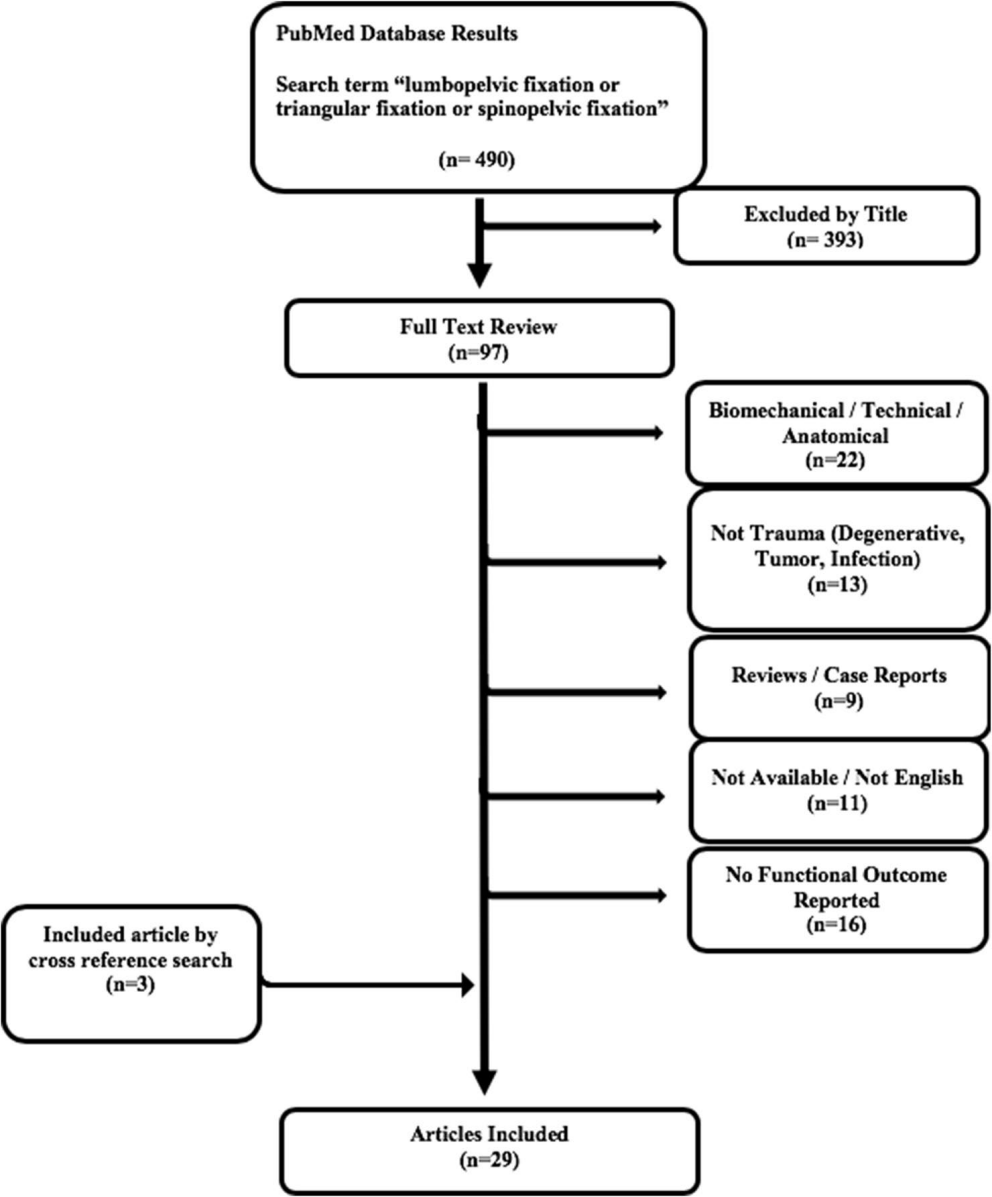
Flow diagram (“Literature review” section) “Lumbopelvic Fixation” (access date 06/20/2020).

This search resulted in 490 articles, of which 393 were excluded by title. After a full text review, a total 29 studies reporting functional outcome after lumbopelvic fixation remained (Fig. 2).

Of the 29 studies, 25 were retrospective cohort analysis.

The cohort size throughout all studies was small, with a total number of included patients of 401 (223m/78f), averaging 17.2 patients per study with an average age of 34.1 years and an average follow-up of 26.5 months (Table 2).

**Table 2:** Mastersheet Literature Review on Functional Outcome in Lumbopelvic Fixation.

Author (year)/journal	Technique	Objective	Study design	Sample size	Gender	Study population	Mean ISS (range)
Schildhauer et al. (2006)/J Orthop Trauma^5^	LPF	To report results of sacral decompression and lumbopelvic fixation in neurologically impaired patients with highly displaced, comminuted sacral fracture-dislocations resulting in spino-pelvic dissociation	rca	n = 19	11m/8f	Highly displaced comminuted, irreducible Roy-Camille type 2-4 sacral fractures with spinopelvic instability and cauda equina deficits	n/a
Bellabarba et al. (2006)/Spine^6^	LPF	To review the safety and patient impact of early surgical decompression, and rigid segmental stabilization in patients with high-grade sacral fracture dislocations	rca	n = 19 (11m/8f)	11m/8f	19 patients with Denis zone 3 injuries. Six presented with Roy-Camille type 3 injuries, 4 with type 4 injuries, and 9 with type 2 injuries. Two fractures were open secondary to extensile perianal soft tissue lacerations. There were 10 other patients who were considered to have clinically relevant soft tissue contusions with lumbodorsal fascial degloving analogous to the Morel Lavalle lesion	3 pat. with postop. infections ISS > 20 versus non-infection ISS 14
Lindahl (2008)/Suomen Ortopedia ja Traumatologia^a^^25^	LPF	To describe the functional outcome in patients with spinopelvic dissociation	rca	n = 19	8m/11f	Patients with spinopelvic dissociation and type 2–3 Roy-Camille + Strange-Vognsen/Lebech fractures; bilateral vertical sacral fractures with spinopelvic instability and cauda equina deficits and/or lumbosacral plexus injury	40 (18–66)
Lindahl (2009)/Suomen Ortopedia ja Traumatologia ^a^^26^	LPF	To evaluate the results of operative reduction and lumbopelvic fixation of patients with high-energy sacral fracture dislocations with spino-pelvic dissociation and neurologic deficits	**pca**	n = 22	10m/12f	Patients with Roy-Camille type 2 or type 3 comminuted bilateral vertical and horizontal sacral fractures with spinopelvic instability and cauda equina deficits and/or lumbosacral plexus injury, were treated with segmental lumbopelvic fixation	41 (18–66)
Gribnau et al. (2009)/Injury^13^	Different methods of posterior stabili-zation	This study intended to assess the injury characteristics, choice of treatment and quality of life of U-shaped sacral fractures	rca	n = 8	3m/5f	Patients with a high-grade U-shaped sacral fracture (Denis Zone III) were included in the study. All patients suffered high-energy trauma. Mechanism of injury included suicidal leaps (n = 7) and accidental falls from heights (n = 1). The fall height ranged from 10 to 20 m.The method of fixation was individualised and consisted of either open posterior transsacral plate fixation, percutaneous sacroiliac screw fixation or open triangular lumbosacral fixation	23 (17–45)
Sagi et al. (2009)/J Orthop Trauma^7^	LPF	To analyze the radiographic, clinical, and functional results of triangular osteosynthesis constructs for the treatment of vertically unstable comminuted transforaminal sacral fractures	**pca**	n = 40	n/a	Patients with vertically unstable pelvic injuries were treated with triangular osteosynthesis fixation	n/a
Jones et al. (2012)/Clin Orthop Relat Res^10^	LPF	To assess the reduction quality and loss of fixation, pain related to prominent hardware, subjective dysfunction measured by the Short Musculoskeletal Function Assessment (SMFA), and complications	rca	n = 15	7m/8f	Patients with unstable sacral fractures treated with lumbopelvic fixation	4/15 patients had an ISS > 15 and were classified as polytrauma
Tan et al. (2012)/Injury^27^	LPF	To report the outcome of patients who underwent lumbopelvic fixation for spinopelvic instability	rca	n = 9	6m/3f	Patients with spinopelvic instability and cauda equina deficits; the vertical fractures totally involved zone II of the sacrum, and most were comminuted	n/a
Ayoub (2012)/Eur Spine J^18^	LPF	To evaluate and analyze the results of surgical decompression and lumbopelvic fixation of these injuries	rca	n = 28	17m/11f	Patients with displaced spinopelvic dissociation and cauda equina syndrome Roy-Camille classification: Type 2: 13 Type 3: 15; Cauda equina syndrome: incomplete: 17 complete: 11; Unilateral L5–S1 facet joint injury: 13; Direct decompression: 14 Indirect decompression: 14	n/a
Hu et al. (2013)/Eur Spine J^28^	LPF	The aim of this study was to explore the operative technique and effectiveness of triangular osteosynthesis for vertically unstable sacral fractures	**pca**	n = 25	12m/9f	13 mva, 6fall from height; 16 cases of unilateral vertical unstable sacrum fractures were fixed with unilateral triangular osteosynthesis; 3 patients with bilateral sacrum fractures were fixed with bilateral triangular osteosynthesis; 3 bilateral fractures were fixed with unilateral triangular osteosynthesis as one side of the sacrum fracture was stable; 5 patients were performed sacral laminectomy for cauda equina decompression; 8 patients who suffered sacral plexus impairment were decompressed through fracture reduction or their small fractures were removed	n/a
Dalbayrak et al. (2013)/Turk Neurosurg^29^	LPF	To describe the outcome of standard lumboiliac instrumentation in patients with spinopelvic instabilities	rca	n = 10	6m/4f	Denis type 1: 4 Denis type 2: 3 Denos type 3: 2 unilateral sacroiliac instability: 6 bilateral sacroiliac instability: 4	n/a
He et al. (2014)/Orthopedics^30^	LPF	To report the authors’ experience with treating patients with type III Denis sacral fracture with lumbopelvic dissociation	rca	n = 21	13m/8f	Fall: 13 traffic trauma: 6 crush-related injury: 2 involved multiple injuries:11; Roy-Camille classification: Type 2: 9 Type 3: 12	n/a
Lindahl et al. (2014)/Injury^19^	LPF	The aim of this retrospective study was to evaluate the radiological and clinical outcomes including neurological recovery after segmental lumbopelvic fixation of spinopelvic dissociation, as well as to uncover prognostic factors of outcome	rca	n = 36	18m/18f	fall from a height: 27 mva: 6 crush injury: 3 median fall height was 10 m (range, 2–20 m); 12 patients had concomitant fractures; All 36 patients had AO type C3 pelvic injuries and Denis zone III H-shaped sacral fractures. Roy-Camille classification: Type 2: 15 Type 3: 21 16 patients had complete translational displacement in the transverse sacral fracture in either ventral or dorsal direction	27 (16–54)
Williams et al. (2016)/J Orthop Trauma^31^	Percu-taneous LPF	To describe a percutaneous lumbopelvic reduction and fixation technique to reduce complications	**pca**	n = 17	n/a	Bilateral longitudinal and transverse sacral fracture patterns (U/H-tpye)	n/a

Author (year)/journal	Follow-up	SMFA	SF-36	EQ-5D/EQ-6D	ODI	Pelvis outcome socre	Pain
Schildhauer et al. (2006)/J Orthop Trauma ^5^	Average 31 mo (12–57)	n/a	n/a	n/a	n/a	n/a	n/a
Bellabarba et al. (2006)/Spine^6^	Average 25 mo (7–37)	n/a	n/a	n/a	n/a	n/a	Average VAS 5.5
Lindahl (2008)/Suomen Ortopedia ja Traumatologia ^a^^25^	n/a	n/a	n/a	n/a	n/a	Mean Hannover score: 5,3 (3–7) postoperatively	n/a
Lindahl (2009)/Suomen Ortopedia ja Traumatologia ^a^^26^	n/a	n/a	n/a	n/a	n/a	n/a	n/a
Gribnau et al. (2009)/Injury^13^	36 mo (5–36)	n/a	n/a	Median EQ-6D VAS score was 70 (range, 50–80)	n/a	n/a	n/a
Sagi et al. (2009)/J Orthop Trauma^7^	18 mo (12–23)	Function and daily activity showed significant improvements; function index improved from an average of 26 at 6 mo to 21 at 1 year (*p* = 0.07); bother index improved from 29 at 6 months to 24 at 1 year (p = 0.17); daily activity index improved from 33 to 23 (p = 0.01); mobility improved from 33 at 6 months to 29 at 1 year (*p* = 0.17)	SF-36v.2 physical component scores averaged 42 (range 26.6–62.8) at 6 months and 46 (range 31.6–63) at 1 year	n/a	n/a	n/a	n/a
Jones et al. (2012)/Clin Orthop Relat Res^10^	23 mo (12–41)	11/15 patients were able to return to work or activities. 4/15 patients had palpable prominent posterior hardware. 4 patients had associated lower extremity injuries, which did not affect daily activity, mobility, dysfunction, or bother at any time	n/a	n/a	n/a	n/a	Greater pain at 1 year in patients with prominent hardware (3.5 out of 5) compared with patients without prominent hardware (1.75 out of 5)
Tan et al. (2012)/Injury ^27^	21.7 mo (14–32)	n/a	n/a	n/a	n/a	n/a	n/a
Ayoub (2012)/Eur Spine J^18^	26 mo	n/a	n/a	n/a	n/a	Excellent: 5 Good:14 Fair: 7 Poor: 2	n/a
Hu et al. (2013)/Eur Spine J^28^	14 mo (8–26)	n/a	n/a	n/a	n/a	n/a	n/a
Dalbayrak et al. (2013)/Turk Neurosurg ^29^	39.2 mo (6–91)	n/a	n/a	n/a	Preoperative ODI: 91.2; postoperative ODI: 24.4	n/a	Preoperative VAS: 8.4; postoperative VAS: 2.2
He et al. (2014)/Orthopedics ^30^	20 mo (8–36)	n/a	n/a	n/a	n/a	n/a	n/a
Lindahl et al. (2014)/Injury^19^	33 mo (18–71)	n/a	n/a	n/a	n/a	n/a	n/a
Williams et al. (2016)/J Orthop Trauma ^31^	21 mo	n/a	n/a	n/a	n/a	n/a	

Author (year)/journal	Technique	Objective	Study design	Sample size	Gender	Study population	Mean ISS (range)
De lure (2016)/Injury^32^	LPF +trans-verse bar	To analyze short- and long-term complications and final clinical outcome in this series	rca	n = 11	6m/5f	11 patients with severe posttraumatic lumbopelvic instability following a high-energy trauma	33.7 (17–50)
Yu et al. (2016)/Injury^33^	LPF	To report the peri-operative results and surgical outcomes of patients with vertical unstable sacral fractures who underwent lumbopelvic fixation through a modified subcutaneous route for iliac screw fixation	rca	n = 28	8m/19f	28 consecutive patients with vertical unstable sacral fractures Fall from height: 15 Motor vehicle collision: 12 Blunt trauma: 1 Roy-Camille Classification: Type 1: 6 Type 2: 9 Type 3: 1 Type 4: 3	19.5 (9–50)
Piltz et al. (2017)/Eur Spine J^34^	mod. LPF	Purpose of this study is to present a surgical technique that facilitates the reduction and the stabilization of these injuries	rca	n = 3	3f	Fall, suicidal fall, mva; Roy-Camille classification: 2,4,1; neurological deficit: sensory deficit root S1&S2, none, sensory deficit root S1 right side	n/a
Jazini et al. (2017)/The Spine Journal^35^	MIS LPF	The study aimed to determine whether minimally invasive LPF provides reliable frac- ture stability and acceptable complication rates in cases of complex sacral fractures	rca	n = 24	12m/12f	“24 patients who underwent MISLPF for complex sacral fracture with or without associated pelvic ring injury; mva: 11 Falls: 6 sacral fracture morphologies: vertical (Zone I, 4 of 24 injuries; Zone II, 7 of 24; Zone III, 2 of 24), transverse (12.5%; Zone III, 3 of 24) H-type (16.7%; Zone III, 4 of 24) T-type (8.3%; Zone III, 2 of 24) U-type (4.2%; Zone III, 1 of 24) lambda-type (4.2%; Zone III, 1 of 24) Out of the Denis Zone III injuries (n = 13) five were Roy-Camille type 1, four were type 2, three were type 3, and one was type 4. bilateral LPF (22 of 24 constructs) with instrumentation from L5 to the ilium (17 of 24 constructs)	27 (5–48)
Xie et al. (2018)/Current Medical Science^14^	LPF	To examine the use of lumbopelvic fixation or (and) sacral decompression to treat U-shaped sacral fractures and the quality of life of patients after treatment in an attempt to provide evidence of effects of such treatments on functional and neurological recovery of patients with U-shaped sacral fractures	rca	n = 15	9m/6f	Consecutive patients with U-shaped sacral fractures; all high energy traumas; most patients underwent LPF and sacral decompression	28.2 (20–43)
Nonne et al. (2018)/J Med Case Rep^36^	LPF + trans-verse bar	To report 5 cases of patients with spinopelvic dissociation	rca	n = 5	2m/3f	Five patients with spinopelvic dissociation. All patients showed severe neurologic lesions: cauda equina syndrome (n = 3) and bilateral radicular L5–S1 deficit (n = 4). Roy-Camille II n = 2; III n = 2; IV: n = 1. One patient died	n/a
Tian et al. (2018)/Orthopaedic Surgery^37^	mod. LPF	To evaluate the clinical outcomes of traumatic spino‐pelvic dissociation (TSD) treated with modified bilateral triangular fixation	rca	n = 18	14m/4f	Falling: 16 mva: 2 all sacral fractures had associated injuries U‐shaped fractures: 10 H‐shaped fractures: 6 Y‐shaped fractures: 2 Roy–Camille classification: type II: 12 type III: 6 sacral plexus decompression: 6 cases	n/a
Futamura et al. (2018)/ International Orthoaedics ^38^	mod. LPF	To describe the procedure and outcomes of a new approach, which we refer to as “within ring”-based sacroiliac rod fixation (SIRF)	rca	n = 15	10m/5f	Fall:7 mva: 5 Compression by a heavy item:3 AO/OTA class: 61-B2.3: 1 C1.3: 4 C2.3: 7 C3.3: 1 H-type spinopelvic dissociation: 2	16.9 (9–30)
Chou et al. (2018)/Journal of the American Academy of Orthopaedic Surgeons ^39^	MIS-LPF	To present a series of spinopelvic dissociation cases from a level I trauma center	rca	n = 18	n/a	None of the patients underwent open spinal surgical decompression	n/a
Abo-Elsoud et al. (2018)/Journal of Orhtopaedic Trauma^40^	mod.LPF	To preset a modified biplanar posterior pelvic fixation technique in patients with unstable sacral fractures	rca	n = 16	9m/7f	Patients with unilateral vertical sacral fractures showing fracture comminution, gaps, vertical instability, and/or disruption of the L5/S1 facet joint	n/a
Shah et al. (2019)/Cureus^41^	MIS-LPF	To analyze the outcome and complications of patients who underwent minimally invasive lumbopelvic fixation to treat unstable U-type sacral fractures	rca	n = 10	n/a	Adult patients with U-type or vertical shear fractures	n/a
Santoro et al. (2019)/World Neurosurgery ^42^	Navigated Spino-pelvic and Sacro-pelvic Stabili-zation	To analyze the difficulties and advantages for surgeons by using digital navigation based on preoperative computed tomography	rca	n = 25	21m/4f	Adults patients with pelvic fractures (Tile classification): B1 n = 5; B2 n = 2; B3 n = 2; C1 n = 9; C2 n = 3; C3 n = 4	n/a
Korovessis et al. (2019)/European Spine Journal^43^	LPF	To evaluate the efficacy and safety of contemporary spinal instrumentation for AO C-type posterior pelvic ring injuries	rca	n = 6	4m/2f	Patients with AO C-type posterior pelvic ring injuries: C1 n = 1; C2 n = 2; C3 n = 3	n/a
Kanezaki et al. (2019)/Medicine^44^	Minimal invasive LPF	To describe the minimal invasive technique and report the preliminary clinical results	rca	n = 10	6m/3f	Denis Zone 1 n = 2 Denis Zone 2 n = 2 Denis Zone 3 n = 6 Roy-Camille classification type 1 n = 4 type 2 n = 2	n/a
Kelly et al. (2018)/Journal Spine Surgery ^24^	LPF	To compare surgical outcomes of U and H type sacral fractures with surgical management by lpf (or iliosacral screw fixation)	rca	n = 8	n/a	Roy-Camille classification (mean): 2.1 Seven out of eight patients underwent sacral decompression	n/a

Author (year)/journal	Follow-up	SMFA	SF-36	EQ-5D/EQ-6D	ODI	Pelvis outcome socre	Pain
De lure (2016)/Injury^32^	7.2 y (4–13.2 y)	n/a	n/a	n/a	2 patients with minimal disability, four with moderate disability, three with severe disability, and none crippled or above 80% of the index	n/a	Light-to-moderate lower back pain: 6 night-time pain: 2 at final follow-up one patient reported only light pain following intense physical activity
Yu et al. (2016)/Injury^33^	12 mo	n/a	n/a	n/a	n/a	n/a	n/a
Piltz et al. (2017)/Eur Spine J^34^	47, 33, 29 mo	n/a	n/a	n/a	n/a	n/a	n/a
Jazini et al. (2017)/The Spine Journal^35^	18.8 mo (0.4–64)	n/a	n/a	n/a	n/a	n/a	n/a
Xie et al. (2018)/Current Medical Science^14^	22.7 mo (9–47)	n/a	n/a	EQ-5D preop: mean 0.203 (0.144–0.279) versus EQ-5D postop mean 0.786 (0.636–0.,819)	n/a	n/a	All patients reported pain: average preop VAS score of 7.07 (5–9) postoperative VAS score of 1.93 ( 1–3) (*p *< 0.0001)
Nonne et al. (2018)/J Med Case Rep^36^	20 mo (12–36)	n/a	n/a	n/a	n/a	n/a	n/a
Tian et al. (2018)/Orthopaedic Surgery^37^	32.4 mo (22–48)	n/a	n/a	n/a	n/a	n/a	n/a
Futamura et al. (2018)/International Orthoaedics^38^	23.8 mo (4–50)	n/a	n/a	n/a	n/a	n/a	n/a
Chou et al. (2018)/Journal of the American Academy of Orthopaedic Surgeons ^39^	18mo (12–68)	n/a	n/a	EQ-5D-5L: 6 patients unable to contact, two remained homeless with no contact details, one patient was sectioned in a mental health unit, 3 were lost to follow-up 2 retired from work because of age,2 remained homeless and unemployed 5 have returned to full work	n/a	n/a	n/a
Abo-Elsoud et al. (2018)/Journal of Orhtopaedic Trauma^40^	29.5mo (14–43)	n/a	n/a	n/a	n/a	n/a	3 Patients requested implant removal because of implant prominence (1 patient) or lower lumbar pain (2 patients)
Shah et al. (2019)/Cureus^41^	2–3 months	n/a	n/a	n/a	n/a	n/a	VAS 1.7 at follow-up
Santoro et al. (2019)/World Neurosurgery ^42^	Mean fu 12 months	n/a	n/a	n/a	n/a	n/a	n/a
Korovessis et al. (2019)/European Spine Journal^43^	61 ± 8 months	n/a	n/a	n/a	n/a	n/a	n/a
Kanezaki et al. (2019)/Medicine^44^	15.0 ± 8.5 months	n/a	n/a	n/a	n/a	n/a	n/a
Kelly et al. (2018)/Journal Spine Surgery^24^	18 mo (1–52)	n/a	n/a	n/a	n/a	n/a	n/a

*n/a* not applicable, *ISS* inury severity score, *mo* months, *rca* retrospective cohort analysis, *pca* prospective cohort analysis, *OR* operation room, *VAS* visual analoge scale, *mva* motor vehicle accident, *fu* follow-up.

^a^Not currently indexed for MEDLINE.

## Discussion

The aim of this study was to analyze functional outcomes after lumbopelvic fixations in patients with traumatic instabilities and discuss the results in the context of the existing literature.

### SMFA

Using the SMFA, Jones et al. analyzed 15 patients (mean age 39 years, follow-up 23 months; 4/5 ISS > 15) with unstable sacral fractures treated with lumbopelvic fixation and found long-term dysfunction compared with normative SMFA. The permanent dysfunction and bother index sub scores were similar to chronic spinal disorders and lower extremity osteoarthritis^10^. By comparison, our results found higher scores over almost all sub-scores, suggesting a higher impairment. This might be explained by the distinctly higher mean age (60 vs. 39 years) in our cohort. Interestingly, in our cohort we found a significant higher impairment (daily activities, mobility, function) in patients suffering from a low-energy trauma compared to patients suffering from a high-energy trauma. This might also be explained by the significantly higher age (72.2 vs. 53.8 years; *p* = 0.030) of the low-energy group. The only other study evaluating functional outcome after lumbopelvic fixation using the SMFA questionnaire was reported by Sagi et al. The author treated 40 patients (mean age 39 years, follow-up 18 months) with vertically unstable pelvic injuries. The indexes improved from 6-months to the 12-months follow-up, but the majority of the patients were still showing higher impairment compared to the population mean. However, 37 of 40 patients were able to return to work and/or schooling^7^.

### EQ-5D

Mobility is an important factor affecting patient’s daily activities and quality of life^11,12^. There are only few studies reporting quality of life in patients who underwent lumbopelvic fixation. Gribnau et al. reported 8 patients who underwent either lumbopelvic fixation with or without transsacral plating, iliosacral screw fixation, or transsacral plate osteosynthesis. The mean follow-up was 36 months in their study and life quality was measured using the EuroQol-6D. The mean EQ-VAS score was 70 (50–80). The authors reported that mood disorders, pain and mobility were influencing general health status^13^. Xie et al. reported on 15 patients after high-energy trauma with U-shaped sacral fractures (mean age 28.8 years) who underwent lumbopelvic fixation and sacral decompression. At follow-up (22.7 months; 9–47 months) all patients reported pain. The average preoperative EQ-5D was 0.203 (0.144–0.279) and postoperatively 0.786 (0.636–0.819)^14^. As already stated by Gribnau et al., the impact of the operative treatment on the long-term morbidity after unstable sacral fractures is difficult to assess due to often present concomitant injuries in those patients.

### Oswestry disability index (ODI)

The ODI is a valid and widely used outcome measure in the management of spinal disorders^15^. De Lure reported on the use of a modified technique for lumbopelvic fixation in their series of 11 patients with lumbopelvic instabilities. Two patients showed minimal disability, four a moderate disability, and three a severe disability at final follow-up (35.5 months). In their cohort of ten patients with traumatic spinopelvic instabilities, Dalbayrak observed an improved ODI from 91.2 preoperatively to 24.4 at follow-up (39.2 months).

### Majeed score/hannover pelvis outcome score (POS)

The Majeed pelvic score is a non-validated self-reported outcome score assessing five dimensions including standing, pain, work, sitting and sexual intercourse. The reported Majeed scores are mostly favorable ranging from 62 to 86.7. Three studies reported a less favorable outcome in the Majeed score. Nonne et al. reported an average Majeed score of 62. However, it should be noted that three of the five reported patients suffered a spinopelvic dissociation. Lindahl reported two studies with a fair functional outcome. Both studies consisted of polytraumatized patients with an average Injury Severity Score (ISS) of 40 and 41, respectively. For comparison, the average reported ISS among all included studies was 28.5 (Table 3). An association of traumatic spinopelvic dissociations with a high ISS scores has been described before^4,13,16,17^. In our series, all patients with concomitant injuries showed a significant worse outcome in five out of six dimensions (except Arm/Hand score) compared to patients without any associated injuries (Table 1).

**Table 3:** Majeed score/ISS score.

Author (year)	n	Majeed score	Mean ISS
Lindahl (2008)	19	67.9	40
Lindahl (2009)	22	3 (excellent), 10 (good), 2 (fair), 6 (poor)	41
Tan (2012)	9	74.3	n/a
Hu (2013)	25	13 (excellent), 6 (good), 2 (fair), 1 (poor)	n/a
Yu (2016)	28	84.5	19.5
Nonne (2018)	5	62	n/a
Tian (2018)	18	12 (excellent), 4 (good) 2 (fair)	n/a
Futamura (2018)	15	86.7	16.9
Abo-Elsoud (2018)	16	9 (excellent), 2 (good), 2 (fair), 2 (poor)	n/a
Korovessis (2019)	6	79 ± 18 (excellent)	n/a
Kanezaki (2019)	10	8 (excellent), 1 (good), 1 (fair)	n/a

The reported Pelvis Outcome Scores ranged from fair (Lindahl et al.) to good (Ayoub et al., Table 2). Ayoub et al. reported a satisfactory outcome in 67.9% of the 28 patients with displaced spinopelvic dissociation and sacral cauda equina syndrome. They analyzed factors affecting the final pelvic outcome using the Pelvis Outcome Score and showed that outcome was significantly better in patients with Roy-Camille type II fractures (vs. type III fractures), road traffic injuries, males, initial transverse fracture kyphosis angle < 40° and a primary direct decompression. Furthermore, they reported significant improvement of sacral fracture kyphosis (58.3° vs. 13.6°, *p* = 0.001), and no loss of reduction was observed at the final follow-up^18^. Lindahl et al. demonstrated a correlation between radiographical results and clinical scores. 74% of patients with “excellent” radiographical results had a good clinical outcome, whereas the majority (59%) of the patients with just a “good” radiographical result had a poor clinical outcome. The authors also observed an association of post-operative kyphosis with the POS. Patients with poor POS had significantly higher kyphosis (29° vs. 17°, *p* = 0.018) compared to patients with good POS^19^.

### Gibbons score/neurological outcome

The Gibbons classification is widely used to assess neurological deficits in patients with sacral fractures and was reported in 15 out of 29 studies^20^. Throughout the analyzed studies, average improvement in the Gibbons classification from pre-operative status to follow-up examination was 1.0 (0.2–1.7; Table 4). Two studies reported significantly lower improvement in their cohorts. Jazini et al. excluded patients who needed open decompression from their study and Futamura’s patients already had very little neurological deficit pre-operatively (1.1).

**Table 4:** Gibbons classification improvements reported in the literature.

Author (year)	n	Gibbons classification mean improvement (pre/post)	Decompression
Schildhauer (2006)	19	1.2 (4.0/2.8)	19/19
Bellabarba (2006)	19	1.2 (4.0/2.8)	19/19
Gribnau (2009)	8	0.9 (4.0/3.1)	1/8
Tan (2012)	9	1.2 (3.5/2.3)	6/0
Ayoub (2012)	28	1.6 (3.1/1.5)	14 (direct), 14 (indirect)
Hu (2013)	25	1.2 (3.0/1.8)	13/25
He (2014)	21	1.6 (3.4/1.8)	21/21
Lindahl (2014)	36	1.0 (3.7/2.7)	n/a
Jazini (2017)	24	0.3 (1.9/1.6)	Patients with the need for open decompression were excluded
Xie (2018)	15	1.7 (3.3/1.6)	14/15
Tian (2018)	18	0.9 (2.5/1.4)	10/18
Futamura (2018)	15	0.2 (1.3/1.1)	n/a
Kanezaki (2019)	10	0.5 (2.5/2.0)	n/a

The current literature is conflicted with respect to the question of surgical timing and the treatment of neurological deficits. Schildhauer et al. could not find an association between the timing of decompression and neurological recovery^5^. Lindahl et al. confirmed these results and showed that laminectomy does not improve bladder or bowel function in patients who underwent decompression^19,21^. Nevertheless, early and adequate fracture realignment, stabilization of the lumbosacral junction, as well as direct and indirect nerve decompression is still considered to be best medical practice. The reported occurrence of nerve injury in U-shaped sacral fractures ranges up to 94.3%^22^. Most authors agree that early surgical decompression, incomplete nerve injury, and stable fixation is related to better neurological results^18,23^. Furthermore, incomplete neurological injuries are more likely end up in full recovery. Schildhauer et al. reported that 36% of patients with one or more disrupted sacral nerve root recovered fully, whereas 86% of patients with non-disrupted nerve roots achieved a complete recovery of bowel and bladder function^5^. In a study by Lindahl et al. analyzing 36 patients with spinopelvic dissociation, permanent neurological deficits were more likely in patients with complete transverse sacral fracture displacement versus patients with incompletely displaced sacral fractures. They concluded the degree of initial translational displacement of transverse sacral fractures determines neurological recovery and clinical outcome^19^. Furthermore, several factors have been found to be not associated with neurological recovery or outcome including fracture type, soft-tissue lesion (Morel-Lavallee), mechanism of injury, surgical decompression, timing of surgery, age, and sex. These results are in contrast to those of Ayoub et al. who reported better outcomes in patients of male gender, road traffic injuries, initial transverse fracture kyphosis angle < 40°, and with a Roy-Camille type II fracture compared to a type III fracture^18^.

Even though other fixation options such as the iliosacral screw fixation (ISF) or the S2 alar iliac (S2AI) screw are useful options, their feasibility is limited in patients with unstable sacral fractures. In addition to the biomechanical advantages of the lumbopelvic fixation technique, which allows early weight-bearing, Kelly et al. showed, in their study comparing ISF vs. LPF in U/H-Type sacral fractures, that the LPF technique is used more often in younger patients and patients with higher Roy-Camille classification^24^. Therefore, we believe LPF and ISF to be synergistic tools, which are often used in different scenarios and patients.

However, reported functional outcomes suggest that patients who underwent lumbopelvic fixation for traumatic instabilities often suffer functional impairment and do not reach normative data again. Neurological deficits have a major impact on patient’s life quality. The severity of reported injury types, as well as the associated injuries in these often polytraumatized patients often do not allow an accurate pre-operative neurological examination. Furthermore, the significance of neurological recovery is questionable since reporting studies are mostly small retrospective cohort analysis with high variability in reported injuries, surgical techniques, and outcome measures^16^. Prospective clinicals trials with long-term follow-up represent an opportunity for further research in this area.

## Limitations

This study has several limitations. The is single center study is of retrospective design and the sample size is relatively small. Therefore, important data points might have been missed and conclusions should be drawn carefully. Lumbopelvic fixation is a technique which has been used for several different indications. Patients with instabilities in this region requiring LPF often suffer from associated injuries after high-energy trauma. Furthermore, the SMFA questionnaire is limited in analyzing lumbopelvic region related impairments. Similar to the SF-36, this questionnaire only allows for general functional impairments to be clearly detected and distinguished. However, other outcome score such as the Hannover pelvis outcome scale (POS) and the Majeed score are non-validated. This is the first study focusing on an analysis of the functional outcome in patients who underwent lumbopelvic fixation for traumatic instabilities.

## Conclusion

Our results suggest that patients with older age and those with concomitant injuries show a greater impairment according to the SMFA score. Several different outcome scores are used in the mostly small retrospective studies and conclusions should be drawn carefully. However, even though mostly favorable functional outcomes were reported throughout the literature, patients still show some level of impairment and do not reach normative data at final follow-up.
